# Low frequency of germline E-cadherin mutations in familial and nonfamilial gastric cancer

**DOI:** 10.1038/sj.bjc.6690308

**Published:** 1999-04

**Authors:** J Stone, S Bevan, D Cunningham, A Hill, N Rahman, J Peto, A Marossy, R S Houlston

**Affiliations:** Section of Cancer Genetics, Institute of Cancer Research, Sutton, SM2 5NG, UK; Section of Medicine, Royal Mardsen Hospital, Sutton SM2 5NG , UK; Section of Epidemiology, Institute of Cancer Research, Sutton, SM2 5NG, UK

**Keywords:** gastric cancer, germline, E-cadherin mutations

## Abstract

Little is known about the relative contributions of genetic and environmental factors to the development of gastric cancer. Mutations in the cell adhesion molecule E-cadherin are recognized to be associated with the development of undifferentiated, diffuse and invasive gastric cancers. A recent study of two gastric cancer families has shown that germline mutations in the E-cadherin gene can be causative (Guilford P et al, *Nature* 1998; **26**: 402–405). We have examined the E-cadherin gene for constitutive mutations in a systematic series of 106 gastric cancer patients, 10 with a family history of the disease and 96 sporadic cases. No pathogenic mutations were observed in any of the 106 patients. The results indicate that germline mutations in E-cadherin will not account for more than 3% of gastric cancers. © 1999 Cancer Research Campaign


*British Journal of Cancer* (1999) **79**(11/12), 1935–1937
© 1999 Cancer Research Campaign
Article no. bjoc.1998.0308

### Low frequency of germline E-cadherin mutations in familial and nonfamilial gastric cancer

**J Stone[1], S Bevan[1], D Cunningham[2], A Hill[2], N Rahman[1], J Peto[3], A Marossy[1] and RS Houlston[1]**

[1]Section of Cancer Genetics, Institute of Cancer Research, Sutton SM2 5NG, UK; [2]Section of Medicine, Royal Mardsen Hospital, Sutton SM2 5NG, UK; and [3]Section of Epidemiology, Institute of Cancer Research, Sutton SM2 5NG, UK

**Summary** Little is known about the relative contributions of genetic and environmental factors to the development of gastric cancer. Mutations in the cell adhesion molecule E-cadherin are recognized to be associated with the development of undifferentiated, diffuse and invasive gastric cancers. A recent study of two gastric cancer families has shown that germline mutations in the E-cadherin gene can be causative (Guilford P et al, *Nature* 1998; **26**: 402–405). We have examined the E-cadherin gene for constitutive mutations in a systematic series of 106 gastric cancer patients, 10 with a family history of the disease and 96 sporadic cases. No pathogenic mutations were observed in any of the 106 patients. The results indicate that germline mutations in E-cadherin will not account for more than 3% of gastric cancers.

**Keywords:** gastric cancer; germline; E-cadherin mutations

Despite a decline in incidence, gastric cancer remains a major cause of death worldwide (Howson et al, 1986). About 10% of gastric cancers are familial but the relative contributions of genetic and environmental factors are poorly understood (Zanghieri et al, 1990; La Vecchia et al, 1992). Over 90% of gastric cancers are adenocarcinomas. These can be classified according to differentiation and by the histomorphological classification of Lauren (1965) which divides tumours into 'intestinal' and 'diffuse'. This appears to reflect differences in the biological basis of the disease. Intestinal tumours are often ulcerating, are associated with intestinal metaplasia of the stomach and are more common in men. Diffuse tumours are poorly differentiated infiltrating lesions which predominate in younger patients and exhibit a sex ratio close to unity (Mecklin et al, 1988; Lo et al, 1996). Furthermore, diffuse cancers are associated with a higher familial risk (Lehtola, 1978), suggesting that genetic factors play a greater role in their development.

Whilst comparatively little is known about gastric carcinogenesis, mutations in the calcium-dependent cell adhesion molecule E-cadherin are associated with the early development of gastric cancer, particularly those which are undifferentiated, diffuse and invasive (Becker et al, 1994; Oda et al, 1994; Muta et al, 1996; Tamura et al, 1996; Shiozaki et al, 1996). A recent study of two families with familial gastric cancer has demonstrated that constitutive mutations in the E-cadherin gene confer susceptibility to the disease (Guilford et al, 1998). In one of the families a frameshift mutation was identified in exon 15 which co-segregated with the gastric cancer and in the second family affected individuals harboured a premature stop codon interrupting exon 13. In order to evaluate the contribution of germline mutations in E-cadherin to the development of gastric cancer we have analysed blood samples from a consecutive series of 106 patients with the disease.

*Received 4 September 1998*
*Revised 8 October 1998*
*Accepted 12 October 1998*

*Correspondence to:* RS Houlston

### PATIENTS AND METHODS

### Patients

EDTA-venous blood samples were obtained from 106 patients attending the Royal Marsden Hospital with a histologically proven diagnosis of gastric adenocarcinoma. All blood samples were obtained with informed consent and local ethical review board approval. A family history was obtained from all patients. Of the 106 patients, 10 reported a family history of gastric cancer. DNA was extracted from blood samples using a standard sucrose lysis method.

### Methods

The search for germline mutations in the E-cadherin gene was performed using conformational specific gel electrophoresis (CSGE). A combination of published and newly-designed oligonucleotides was used to amplify each exon of E-cadherin (including splice sites) specifically in the PCR (Table 1). CSGE was performed as described by Ganguly et al (1993). All samples with bandshifts were sequenced in duplicate and in forward and reverse orientations after re-amplification of the appropriate exon from genomic DNA in the PCR. Purified PCR products were sequenced using the ABI Ready Reaction Dye Terminator Cycle Sequencing kit and the ABI 377 Prism sequencer.

### RESULTS AND DISCUSSION

Prompted by the recent report of two families in which constitutive mutations in the E-cadherin gene predispose to gastric cancer (Guilford et al, 1998), we have examined a consecutive series of 106 patients for germline mutations. The ages and clinical characteristics of the patients are shown in Table 2.

The average age at diagnosis of the patients we studied is lower than in the general population and they are therefore likely to include a higher proportion of genetically susceptible individuals.

**Table 1** Primers and conditions used for PCR amplification of E-cadherin exons; adapted from Berx et al, 1995

| | Primer sequence | Amplicon size (bp) | $T_A$ (°C) | $MgCl_2$ (mM) |
|---|---|---|---|---|
| 1-F | TACGGGGGGCGGTGCTCCGG | 282 | 68 | 2 |
| 1-R | TCACCCGGTTCCATCTAC | | | |
| 2-F | TCACCGGTTCCATCTAC | 378 | 65/55 Td | 2 |
| 2-R | CAACCTCCTCTTCTTTAT | | | |
| 3-F | GCTCTTGTCTTTAATCTGTC | 360 | 65/55 Td | 1.5 |
| 3-R | GTACCAAGGCTGAGAAACCT | | | |
| 4 and 5-F | CTTGTTCCTCATCTTCTTTC | 603 | 65/55 Td | 2 |
| 4 and 5-R | CCCATCACTTCTCCTTAGAGCA | | | |
| 6-F | CTCACTTGGTTCTTTCAG | 246 | 55 | 1.5 |
| 6-R | AACCTTTGGGCTTGGACA | | | |
| 7-F | AGCTTGTCTAAACCTTCATC | 329 | 65/55 Td | 2 |
| 7-R | GCTTAGACCATCACTGTATT | | | |
| 8-F | TTGGTTGTGTCGATCTCTCT | 223 | 60/50 Td | 2.5 |
| 8-R | CAGTGGTACCCTTAGTTCAT | | | |
| 9-F | GTACTTGTAATGACACATCTC | 252 | 55 | 2 |
| 9-R | TGCCAGTTTCTGCATCTTGC | | | |
| 10-F | ACTTCATTGTTTCTGCTCTC | 311 | 65/55 Td | 2 |
| 10-R | AACCAGTTGCTGCAAGTCAG | | | |
| 11-F | GTTGTTTGCTGGTCCTATTC | 253 | 65/55 Td | 2 |
| 11-R | GAACTAGCTAGGAGGTCGAG | | | |
| 12-F | TGGGGATTCATTACTGTTGC | 326 | 65/55 Td | 1.5 |
| 12-R | GCATGGCAGTTGGAGCAAAG | | | |
| 13-F | TTTCCTCCCCTGGTCTCATC | 302 | 65/55 Td | 2 |
| 13-R | TGAGTCACTTGCCAGCTGGA | | | |
| 14-F | CTCTCAACACTTGCTCTGTC | 209 | 65/55 Td | 1.5 |
| 14-R | AGAGATCACCACTGAGCTAC | | | |
| 15-F | CATAGCCCTGTGTGTATGAC | 248 | 65/55 Td | 1.5 |
| 15-R | CGGATGCTTTGGCTTTCCAC | | | |
| 16-F | AGATGACAGGTGTGCCCTTC | 315 | 65/55 Td | 1.5 |
| 16-R | ATTTCTGCATTTCCCAGCAC | | | |
| 4/5–3[a] | CTTTCATTTTGTCTTCAG | | 55 Td | 1.5 |

[a]Used for sequencing of exon 5.

Family histories were obtained from all patients and 10 reported a history of gastric cancer in at least one relative. Details of these familial cases are shown in Table 3. None of the cases studied had family histories indicative of either hereditary nonpolyposis colon cancer (HNPCC) or breast–ovarian cancer syndromes.

We have screened the full coding sequence and splice junctions of E-cadherin for germline mutations in these 10 familial and 96 sporadic gastric cancer patients. No clearly disease-causing mutations in the E-cadherin gene were identified in any of the 106 patients screened. Five variants were detected (Table 4). The two variants in exons 13 and 14 have been previously reported (Risinger et al, 1994; Berx et al, 1995). The polymorphism in exon 13 was a synonymous C–T substitution at position 3 of codon 692 encoding alanine, and the polymorphism in exon 14 was a synonymous C–T substitution at position 3 of codon 751 encoding aspartine. The other three variants detected were in the 5′ untranslated region of exon 1 and have not been reported previously. It is conceivable, but not likely, because none had a family history of cancer, that the mutations we detected in the 5′ untranslated region of the gene might be pathogenic by affecting the expression of E-cadherin either directly or through linkage disequilibrium with other mutations affecting promotor function.

The results suggest that germline mutations in E-cadherin are rare in gastric cancer patients. We cannot exclude the possibility that a minority of mutations have been missed, but under test conditions we have found that CSGE can detect all small insertions or deletions and 90% of single base substitutions, and the technique detected a number of single base substitution polymorphisms within the gene. Based on the number of gastric cancer patients we have screened for constitutive mutations we can conclude with 95% probability that germline variation in E-cadherin will not account for more than 3% of all gastric cancers in the British population.

The recent report demonstrating that constitutional mutations in E-cadherin can confer a high gastric cancer risk was based on two families from Aotearoa, New Zealand (Guilford et al, 1998). Ninety per cent of affected individuals in these families were diagnosed before the age of 65, whereas gastric cancer in the general population is usually diagnosed after the age of 65 and the pattern of inheritance was clearly dominant with a penetrance of around 80% (Guilford et al, 1998). The patients we have studied were from the United Kingdom and were not of comparable age, and none had a family history clearly indicative of a dominant susceptibility gene. Gastric cancers caused by E-cadherin may be restricted to patients with very early onset disease and strong family histories or from specific ethnic groups.

Although mutations in E-cadherin appear to be a rare cause of gastric cancer they provide direct evidence for the existence of a gastric cancer susceptibility syndrome. Prior to the report of Guilford et al (1998) the only inherited forms of gastric cancer

*© Cancer Research Campaign 1999*

**Table 2** Ages and clinical characteristics of gastric cancer patients studied

| | |
|---|---|
| Sex | Male (85): Female (21) |
| Age; years (+/– SD) | |
| All (*n* = 106) | 61.2 (10.6) |
| Positive family history (*n* = 10) | 53.6 (11.0) |
| No family history (*n* = 96) | 62.0 (10.3) |
| Histology | |
| Diffuse | 26 |
| Intestinal | 43 |
| Mixed | 3 |
| Unclassified | 34 |

**Table 3** Details of patients with a family history of gastric cancer

| Index case (age; years) | Relative (age; years) |
|---|---|
| 45 | Father (33) |
| 59 | Uncle (73) |
| 45 | Grandfather (77) |
| 46 | Father (66) and Mother (73) |
| 47 | Mother (80) |
| 75 | Brother (72) |
| 59 | Grandfather (70) |
| 67 | Grandfather (46) |
| 52 | Aunt (29) |
| 41 | Father (45) |

**Table 4** Summary of E-cadherin gene variations detected

| No. | Position (bp) | Polymorphism | Frequency |
|---|---|---|---|
| 2 | – 54; 5′ UTR | G > C | 0.019 |
| 2 | – 71; 5′ UTR | C > G | 0.019 |
| 1 | – 82; 5′ UTR | C > G | 0.009 |
| 54 | Codon 692 | GCC(Ala) to GCT (Ala) | 0.51 |
| 5 | Codon 751 | AAC(Asn) to AAT(Asn) | 0.047 |

recognized were those in association with inherited cancer syndromes such as HNPCC and *BRCA2* (reviewed in Bevan and Houlston, 1999). As E-cadherin appears to be central to the maintenance of gastric mucosa, mutations in genes such as α and β-catenin, which can abolish E-cadherin mediated cell adhesion (Ilyas and Tomlinson, 1997), represent other possible susceptibility genes.

### ACKNOWLEDGEMENTS

We thank the Cancer Research Campaign for support and the patients for their participation in this study. Steven Bevan is in receipt of a Fellowship from the Coeliac Society. Andrea Hill is supported by a grant from the Royal Marsden Hospital Charitable Trust.

### REFERENCES

Becker KF, Atkinson MJ, Reich U, Becker I, Nekarda H, Siewert JR and Hofler H (1994) E-cadherin gene mutations provide clues to diffuse type gastric carcinomas. *Cancer Res* **54**: 3845–3852

Berx G, Cleton-Jansen AM, Nollet F, de Leeuw WJ, van de Vijver M, Cornelisse C and van Roy F (1995) E-cadherin is a tumour/invasion suppressor gene mutated in human lobular breast cancers. *EMBO J* **14**: 6107–6115

Bevan S and Houlston RS (1999) Genetics of gastric cancer. *Q J M* (in press)

Ganguly A, Rock MJ and Prockop DJ (1993) Conformation-sensitive gel electrophoresis for rapid detection of single-base differences in double stranded PCR products and DNA fragments: evidence for solvent-induced bends in DNA heteroduplexes. *Proc Natl Acad Sci USA* **90**: 10325–10329

Guilford P, Hopkins J, Harraway J, McLeod M, McLeod N, Harawira P, Taite H, Scoular R, Miller A and Reeve AE (1998) E-cadherin germline mutations in familial gastric cancer. *Nature* **26**: 402–405

Howson CP, Hiyama T and Wynder EL (1986) The decline in gastric cancer: epidemiology of an unplanned triumph. *Epidemiol Rev* **8**: 1–27

Ilyas M and Tomlinson IPM (1997) The interactions of APC, E-cadherin and β-catenin in tumour development and progession. *J Pathol* **182**: 128–137

Lauren P (1965) The two histological main types of gastric carcinoma: diffuse and so-called intestinal type carcinoma. An attempt at a histoloclinical classification. *Acta Pathol Microbiol Scand* **64**: 31–49

La Vecchia C, Negri E, Franceschi S and Gentile A (1992) Family history and the risk of stomach and colorectal cancer. *Cancer* **70**: 50–55

Lehtola J (1978) Family study of gastric carcinoma; with special reference to histological types. *Scand J Gastroenterol Suppl* **50**: 53–54

Lo SS, Wu CW, Hsieh MC, Kuo HS, Lui WY and P'Eng FK J (1996) Relationship between age and clinical characteristics of patients with gastric cancer. *Gastroenterol Hepatol* **11**: 511–514

Mecklin JP, Nordling S and Saario I (1988) Carcinoma of the stomach and its heredity in young patients. *Scand J Gastroenterol* **23**: 307–311

Muta H, Noguchi M, Kanai Y, Ochiai A, Nawata H and Hirohashi S (1996) E-cadherin gene mutations in signet ring cell carcinoma of the stomach. *Jpn J Cancer Res* **87**: 843–848

Oda T, Kanai Y, Oyama T, Yoshiura K, Shimoyama Y, Birchmeier W, Sugimura T and Hirohashi S (1994) E-cadherin gene mutations in human gastric carcinoma cell lines. *Proc Natl Acad Sci USA* **91**: 1858–1862

Risinger JI, Berchuck A, Kohler MF and Boyd J (1994) Mutations of the E-cadherin gene in human gynecologic cancers. *Nat Genet* **7**: 98–102

Shiozaki H, Oka H, Inoue M, Tamura S and Monden M (1996) E-cadherin mediated adhesion system in cancer cells. *Cancer* **77** (Suppl): 1605–1613

Tamura G, Sakata K, Nishizuka S, Maesawa C, Suzuki Y, Iwaya T, Terashima M, Saito K and Satodate R (1996) Inactivation of the E-cadherin gene in primary gastric carcinomas and gastric carcinoma cell lines. *Jpn J Cancer Res* **87**: 1153–1159

Zanghieri G, Di Gregorio C, Sacchetti C, Fante R, Sassatelli R, Cannizzo G, Carriero A and Ponz de Leon M (1990) Familial occurrence of gastric cancer in the 2-year experience of a population-based registry. *Cancer* **66**: 2047–2051

© Cancer Research Campaign 1999
